# Ag/AgO Nanoparticles Grown via Time Dependent Double Mechanism in a 2D Layered Ni-PCP and Their Antibacterial Efficacy

**DOI:** 10.1038/srep44852

**Published:** 2017-03-21

**Authors:** Rashmi A. Agarwal, Neeraj K. Gupta, Rajan Singh, Shivansh Nigam, Bushra Ateeq

**Affiliations:** 1 Department of Chemistry, Indian Institute of Technology Kanpur, 208016, India; 2 Department of Mechanical Engineering, Indian Institute of Technology Kanpur, 208016, India; 3 Department of Biological Science and Bioengineering, Indian Institute of Technology Kanpur, 208016, India

## Abstract

A simple synthesis route for growth of Ag/AgO nanoparticles (NPs) in large quantitative yields with narrow size distribution from a functional, non-activated, Ni (II) based highly flexible porous coordination polymer (PCP) as a template has been demonstrated. This template is a stable storage media for the NPs larger than the pore diameters of the PCP. From EPR study it was concluded that NPs were synthesized via two mechanisms i.e. acid formation and the redox activity of the framework. Size range of Ag/AgO NPs is sensitive to choice of solvent and reaction time. Direct use of Ag/AgO@Ni-PCP shows influential growth inhibition towards *Escherichia coli* and the pathogen *Salmonella typhimurium* at extremely low concentrations. The pristine template shows no cytotoxic activity, even though it contains Ni nodes in the framework.

Porous coordination polymers (PCPs)/metal organic frameworks (MOFs) are of research interest as they possess structural regularity, high porosity and surface area, and structural transformation ability, thus creating great potential in molecular adsorption and separation processes^1^,^2^, ion exchange^3^, catalysis^4^,^5^, sensor technology^6^, optoelectronics and biomedical applications^7^, etc. These can be either rigid or flexible. Flexible PCPs are more versatile as their frameworks can respond to external stimuli^8^, a unique characteristic, which is applicable for the efficient development of certain devices and sensors. These characteristics can be enhanced by functionalization on both the organic and inorganic parts of the structures^9^. Enhanced functionalization has enabled PCPs to emerge as a new class of templates that combine the long-range order of hard templates with small diameter and synthetically flexible environment of soft templates, thus providing a confined space for nucleation of nanoparticles (NPs). NPs have substantial impact across diverse fields, including catalysis^10^, sensing^11^, photochemistry^12^, optoelectronics^13^,^14^, energy conversion^15^ and medicine^16^.

Depending on both structure and functionality of PCPs, monodispersion to narrow size range NPs can be produced. The physical and chemical properties of metal nanoparticles (M NPs) are essentially divergent from their bulk metals, and are manifested by delocalization of free electrons^17^. The high surface-area-to-volume ratio of NPs provides a large number of active sites, the size and shape of which control reactivity^18^, whereas high surface energy and large surface area of NPs results in drastic reduction of thermodynamic stability which hinders their size, shape and degree of uniformity.

Nucleation includes methodologies such as chemical vapour deposition^19^, solution infiltration with^20^ and without photo-irradiation, solid grinding^21^ and polyol mediated method^22^ of synthesis for introducing metal precursors. Fischer *et al*. investigated both vapor-phase^23^ and solution methods to infiltrate metal organic frameworks (MOFs) with Pd, Pt, Au, Cu, and Ru. As an example, in MOF-5, they obtained Pd, Cu, Au and Ru NPs in the size range of 1.4, 1–4, 5–20, 1.5–1.7 nm respectively after treatment of the inclusion compounds with H_2_ gas^24^. Wang et al. synthesized Pt NPs (2–3 to 5–6 nm) via photo-irradiation of MOFs immersed in precursor solution^20^. Xu and co-workers applied solid grinding methodology to grow Au NPs in MOF-5 from an organovolatile precursor in a stream of 10% volume H_2_ in N_2_ at 230 °C for 2–3 h^21^. A recent method for conversion of MOFs into metal/metaloxide@carbon(M/MO@C) composites with well-defined shapes has been studied by Woojeong *et al*. By this method, porosity of carbon and M/MO particle sizes were controlled by a two-step process that included impregnation of the polymer precursors in vapour form, and condensation polymerization of precursors followed by thermolysis^25^. These cited methods appear to require either higher energy inputs or secondary synthesis steps for the growth of NPs.

Amongst PCPs, redox-active frameworks^26^,^27^,^28^ are useful even though they are extremely rare, as they oxidize or reduce certain metal precursors and include them within their structures. Suh *et al*., have synthesized redox active Ni(II) based PCPs for the growth of Ag and AuNPs (~3–4 and ~2 nm respectively) nucleated initially within the framework which later diffused onto the surface and aggregated with complete or partial dissociation of the framework^26^.

Here we report a highly flexible, layered **Ni-PCP** to nucleate and grow Ag/AgO NPs. It is suggested that both the open flexible structure and functional electronic environment which consist of monodentate binding mode of each carboxylate of BTC colinker which is a very positive site for anchoring metal precursor along with presence of confined sized cavities (in which once NPs are synthesized they will not get agglomerate) within the framework present an excellent opportunity to explore this PCP as a host template to fabricate Ag/AgO NPs without any external reducing agent or secondary steps. An interesting aspect explored is that the Ag/AgO NPs can be extracted without destroying the host framework. It is reported for the first time the potential of using the **Ag/AgO@Ni-PCP** directly for antivirulent properties without the need of extracting the NPs prior to use.

## Results

**Figure 1:**
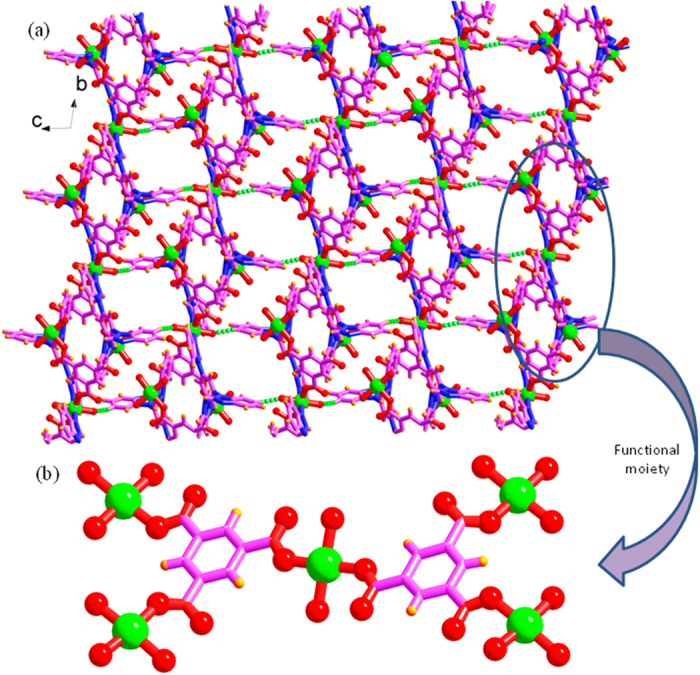
Two dimensional layered crystal structure of porous host template (Ni-PCP). (**a**) Showing three different types of cavities to grow NPs in the **Ni-PCP**. (**b**) Highly functional part of the structure involving free oxygens of each carboxylate groups of BTC linker bonded in a monodentate fashion act as anchoring sites for metal ions of metal precursor. Colour scheme: purple, carbon; red, oxygen; blue, nitrogen; green, nickel.

A previously synthesized **Ni-PCP**, {[Ni_3_(TBIB)_2_(BTC)_2_(H_2_O)_6_]∙5C_2_H_5_OH∙9H_2_O}_n_ [TBIB = 1,3,5-tri(1H-benzo[d]imidazol-1-yl)benzene, H_3_BTC = 1,3,5-benzenetricarboxylic acid)^29^ highly breathing structure at room temperature, comprising of isolated cavities, the largest (diameter ~7.4 Å) containing four ethanol and six lattice water molecules; smaller ones (size ~7.37 × 0.7 Å) containing one ethanol and one water molecule and a macro-cyclic ring (size ~4.6 × 1 Å) within a layer possessing two non-coordinated water molecules was utilized (Fig. 1a) without solvent removal. The functional electronic environment includes free oxygens of each carboxylate groups (BTC molecules) bonded in a monodentate fashion (Fig. 1b). Solvent accessible volume of the PCP is ~36% of the unit cell volume and Brunauer-Emmett-Teller (BET) surface area calculated from CO_2_ sorption at 195 K and one atmospheric pressure, is 297.63 m^2^ g^−1^.

To nucleate and grow Ag/AgO NPs, the **Ni-PCP** was immersed in suitable solution of AgNO_3_ at room temperature for 48 h (See Methods) to afford a brownish fibrous host solid whose morphology is different from the pristine green coloured crystalline polymer (Supplementary Fig. S1). This indicates that there are interactions between the host framework and AgNO_3_.

To check the growth, distribution and location of Ag/AgO NPs, following techniques including powder X-ray diffraction (PXRD), X-ray photoelectron spectroscopy (XPS), electron density spectroscopy (EDS), electron paramagnetic resonance (EPR), zeta potential and high resolution transmission electron microscopy (HRTEM), were employed.

**Figure 2:**
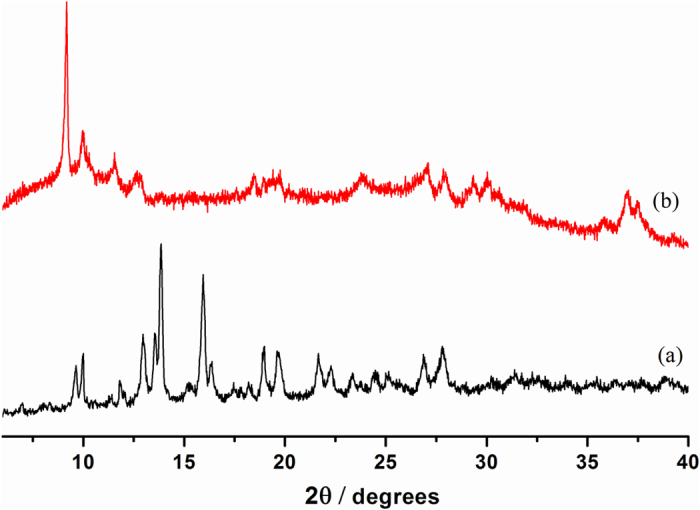
PXRD patterns. (**a**) Host framework (**Ni-PCP**). (**b**) **Ag/AgO@Ni-PCP** showing expansion of the framework after fabrication of NPs in the host which is observed due to slight shifting of peaks towards lower 2θ value.

From the PXRD patterns of **Ag/AgO@Ni-PCP** it is observed that the framework remains intact after generation of Ag/AgO NPs (Fig. 2). Slight peak shifting towards lower 2θ values evidences that the framework has expanded indicating that Ag/AgO NPs are introduced between the host layers. This expansion agrees well with observations of FTIR data (explained later). Crystalline silver appears at 2θ = 37.0° while diffraction peak at 37.5° is due to the presence of AgO which corresponds well with theoretical values of literature^30^,^31^. AgO is formed by the reaction of Ag(0) with O_2_ present in air.

From XPS and EDS data (Supplementary Figs S2 and S3), it is apparent that Ag/AgO and Ni(III) coexist in the framework. In XPS, 3d_5/2_ and 3d_3/2_ occur at 365.6 and 372 eV respectively, which are comparable to standard values (368 and 374 eV) for metallic silver^32^. The binding energy of Ag 3d_5/2_ was shifted to 367.2 eV which indicates the presence of AgO. Further confirmation is obtained from the peaks at 570.4 and 602.4 eV which are ascribed to Ag 3p3 and Ag 3p1 of silver oxide^33^. The peak at 284 and 282.4 eV are attributed to co-condensation of ethanol and methanol on Ni(II) and Ni(III)^34^ in as-synthesized **Ni-PCP** and **Ag/AgO@Ni-PCP** respectively. The peaks at 397.6 and 399.2 eV are assigned to coordinated N-atom of linker on Ni(II) and Ni(III). Peaks at 530.4 eV for both structures are attributed to oxygen of BTC adsorbed on Ni(II) and Ni(III).

Zeta potential measurements shows changes in surface charge of NPs with time. Ag NPs with more positive than +30 mV or negative values more than −30 mV tend to repel each other thus prevents agglomeration and leads to stability for storage^35^. In this case to check zeta potential, suspensions of **Ag/AgO@Ni-PCP** were sonicated for 3 minutes to extract NPs in to the solvent. Three measurements have been taken for two suspensions independently which shows average value of zeta potential is −6.72 to −4.12 mV (Supplementary Fig. S4) showing that these NPs are highly reactive after extraction from the host template.

**Figure 3:**
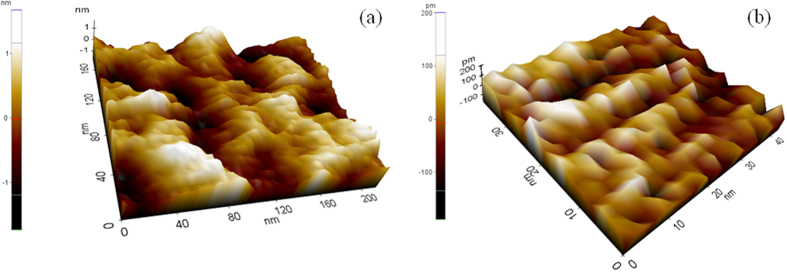
AFM contact mode images. (**a**) Host framework showing open cavities even at a lower resolution. (**b**) Cavities are not visible in **Ag/AgO@Ni-PCP** even at higher resolution due to growth of NPs in a large quantity providing stretched structure.

**Figure 4:**
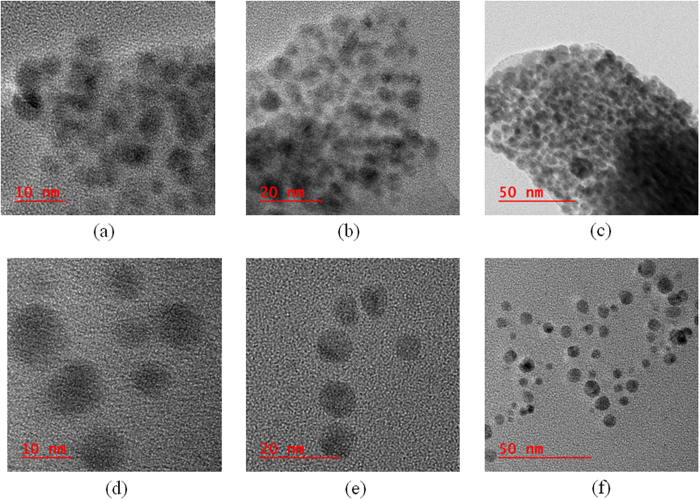
HRTEM images. (**a**–**c)** As-synthesized **Ag/AgO@Ni-PCP** TEM images reveal that NPs are present inside the polymeric membrane of the host. **(d**–**f)** After treating **Ag/AgO@Ni-PCP** with ethanol for 10 minutes, the NPs are released from the framework as shown here.

Contact mode AFM images (Fig. 3) show that the pores of the pristine framework are evenly sized and distributed, although contact mode AFM images do not give very high spatial resolution due to finite size of the tip, and capillary condensation. Surface roughness appears to be in the range of ~2 nm. Images for **Ag/AgO@Ni-PCP** are taken at a much higher magnification as the pores were not visible. The difference between surface topologies of **Ni-PCP** and **Ag/AgO@Ni-PCP** show that the channels after Ag/AgO NPs integration become elongated and narrow with reduction in surface roughness. This confirms that the original structure is now in a highly stretched state due to its inherent flexibility. HRTEM analysis of **Ag/AgO@Ni-PCP** shows the presence of discrete particles with dimensions several times larger (typically 2–8 nm) than those of the host framework cavities. There are no Ag/AgO NPs formed on the surface of the host framework. The images (Fig. 4a–c) show some discrete particles scattered outside the host PCP, which may have been released from the periphery of the structure due to sonication during sample preparation. When synthesis is carried out for an extended period of 96 h, there is very slight agglomeration of particles (Supplementary Fig. S5a and S5b). When the **Ag/AgO@Ni-PCP** is treated with ethanol for 10 minutes, the NPs are released from the framework as seen in HRTEM images (Fig. 4d–f). The reason for this is attributed to swelling of the layered structure in solvent thereby allowing NPs to exit. Also, high mobility as a result of low aggregation and weak attracting forces in the cavities facilitates release of the particles from within the framework. The crystalline planes of NPs are not visible in these images so an image of these NPs where crystalline planes are clearly seen is shown in Supplementary Fig. S6.

The EPR spectrum (Supplementary Fig. S7) of the **Ag/AgO@Ni-PCP** synthesized after 48 h reaction time shows a sharp peak at g = 2.004 indicative of Ag/AgO nanoparticles in a narrow size range with homogeneous distribution. With a reaction time of 96 h, the appearance of a peak at g = 4.36 indicates the presence of octahedral Ni(III) species along with the peak at g = 1.90 with multiple prominent shoulder peaks inferring formation of discrete NPs along with small clusters.

Comparative stabilities of the pristine **Ni-PCP** and **Ag/AgO@Ni-PCP** by thermogravimetric analysis (TGA) (Supplementary Fig. S8) shows that as synthesized PCP undergoes weight loss of 15% between 40–160 °C due to combined evaporation of ethanol and water molecules. This corresponds well with the calculated amount of lattice solvent molecules (17%) (Supplementary Table S1). The host PCP exhibits excellent thermal stability till 420 °C beyond which it undergoes degradation and loss of structure. After formation of Ag/AgO NPs within the framework, thermal stability is drastically reduced and is attributed to the integrated framework being stressed and the catalytic activity of Ag/AgO NPs.

Fourier transform infrared spectroscopy (FTIR) data (Supplentary Fig. S9c) of **Ag/AgO@Ni-PCP** synthesized after 48 h reaction time shows a slight shift of the C-O stretching peak indicating integration of NPs in the framework. **Ag/AgO@Ni-PCP** synthesized after 96 h reaction time shows presence of NO_3_^−^ ions at 1384 cm^−1^ (Supplementary Fig. S9b). Influence of Ag/AgO NPs within the framework is evidenced by noticeable shifting of stretching frequencies towards lower wave numbers. This indicates that due to generation of Ag/AgO NPs, via a double mechanism discussed below the framework is stressed since particles exist in size range of 2–8 nm which exceed the cavity dimensions.

## Discussion

From the above characterizations it is evidenced that there are two mechanisms by which the NPs are synthesized. At a reaction time of 48 h from EPR it is concluded that the single sharp peak of NPs is due to acid formation^36^ in which the NPs are anchored to the free oxygens of carboxylate of the BTC ligand. This mechanism is further supported by FTIR wherein slight peak shifts were observed. Continuing the reaction time up to 96 h, a second mechanism is responsible for further NPs synthesis. EPR spectra of **Ag/AgO@Ni-PCP** shows a peak for Ni(III) which concludes that framework is oxidised. This is further supported by FTIR which shows the presence of NO_3_^−^ anions within the framework to compensate the positive charge of Ni(III). This second mechanism comes in to play when the anchoring sites (free oxygens) are saturated by the NPs. This structure has capacity to store large quantitative yields of Ag/AgO NPs, equivalent to one third of its mass, in spite of low BET surface area.

To check feasibility of synthesizing Ag/AgO NPs in host framework by different solvents namely acetonitrile, water, ethanol and methanol under identical conditions, reactions were performed (48 h). The integrity of the framework was analysed by PXRD which shows that the structures are maintained along with formation of Ag/AgO NPs (Supplementary Fig. S10). In ethanol as reaction media, crysralline silver peak did not appear probably because the nanoparticles are too small to be diffracted. From EPR data of **Ag/AgO@Ni-PCPs** synthesized using different solvents it is seen that Ag/AgO NPs were formed in a narrow size range except in acetonitrile where formation of small clusters are evidenced along with discrete particles. Size range is influenced by molecular radii and polarity of solvent molecules (Supplementary Fig. S11). It is noted from EPR spectrum of **Ag/AgO@Ni-PCP** synthesized using water as solvent homogeneous particle size distribution is obtained due to high polarity which hinders formation of clusters. In the series of solvents used in synthesis, the order of polarity is water > acetonitrile > methanol/ethanol.

**Figure 5:**
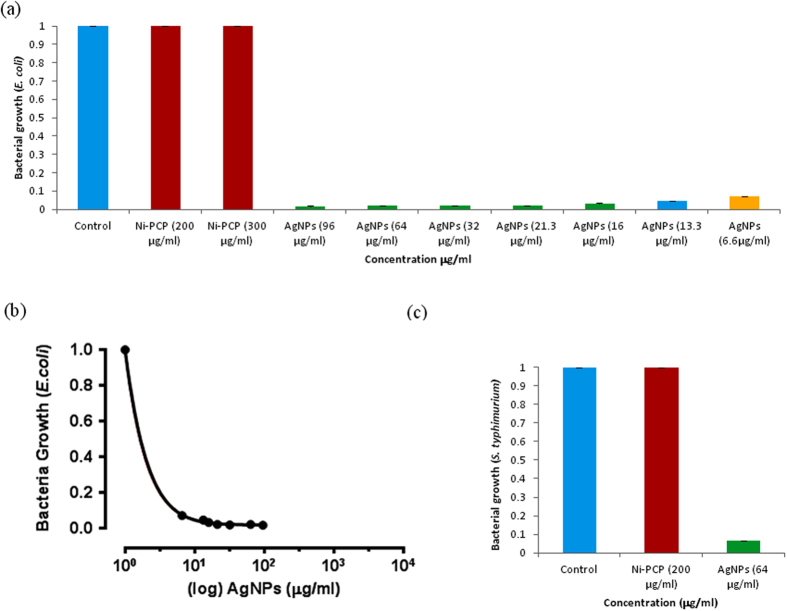
Bacterial growth inhibition curves. (**a**) and (**b**) For *E. coli* no growth inhibition takes place by pristine **Ni-PCP**: Ni in host structure shows no cytotoxicity; **Ag/AgO@Ni-PCP** inhibits growth even at very low concentration 6.6 μg/ml. **(c)** For pathogenic species *S. typhimurium* growth is inhibited due to presence of Ag/AgO NPs exhibiting cytotoxicity.

Ag NPs find biomedical applications in antibacterial paints and disinfectants, surface coating for neurosurgical implements, bone cement and other implants, impregnated wound dressings, etc. However they are prone to oxidation and agglomeration into larger clusters on storage. Herein we report for the first time direct use of **Ag/AgO@Ni-PCP** for growth inhibition of bacteria (*E. coli*) and pathogen (*S. typhimurium*) which are Gram-negative bacteria. It is clearly seen from Fig. 5a that the host framework (**Ni-PCP**) does not display any cytotoxicity despite the presence of Ni in its structure (being fixed in coordination environment). Different concentrations of Ag/AgO NPs present within cavities of **Ag/AgO@Ni-PCP** are released in suspension media under shaking. Sample concentrations of **Ag/AgO@Ni-PCP** used for assay were 300−20 μg/ml corresponding to calculated equivalent of 96−6.6 μg/ml Ag/AgO NPs (See Methods). Complete extraction of Ag/AgO NPs is not expected and the actual concentrations will be still lower than the calculated values stated above. Despite the low concentration of free Ag/AgO NPs in the suspensions, their efficacy in inhibiting bacterial and pathogen growth is strongly evidenced (Fig. 5c). Therefore, it appears that a still lower concentration of **Ag/AgO@Ni-PCP** could possibly be used to inhibit growth under certain conditions. This is an excellent achievement evidenced for this integrated template, not reported elsewhere, and presents potential for biomedical applications.

**Figure 6:**
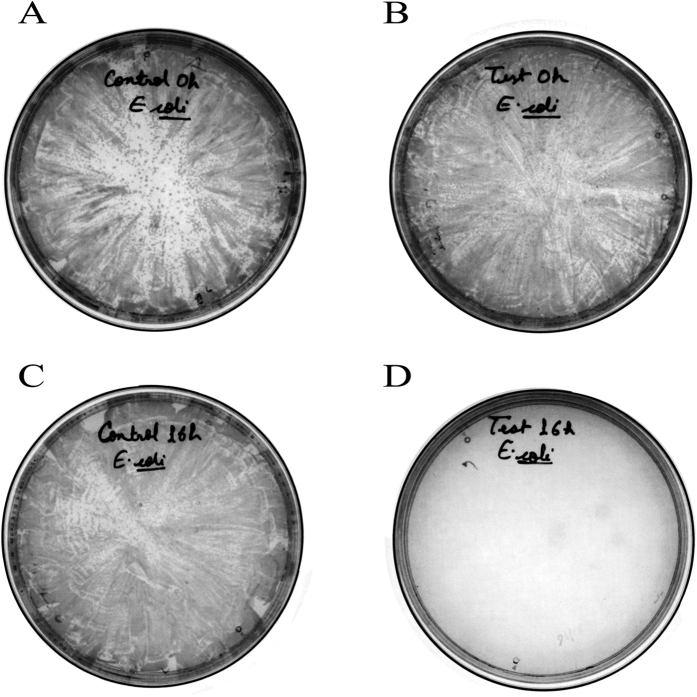
Growth inhibition of *E. coli* DH5α strain by plate culture method at a concentration of 20 μg/ml of Ag/AgO@Ni-PCP for 16 h. While no growth inhibition is seen for 0 h period because during this period no Ag/AgO NPs are extracted from integrated PCP.

There are various factors which may affect the growth of the bacteria, for example nature of nutrients, sterility, growth medium, and other materials present in the medium. So to check these effects on the growth of bacteria with and without NPs another approach classic plates count method has been done, In this experiment (See Methods) a significant growth inhibition was observed by using **Ag/AgO@Ni-PCP** for assay at a concentration of 20 μg/ml for 16 h while no growth inhibition was shown for 0 h period as it is visible in Fig. 6, because during this period no Ag NPs are extracted from integrated PCP.

Antimicrobial efficacy has been shown to be dependent on both size and shape of NPs. Particles that have larger surface areas and come in contact with bacterial cells will have a higher percentage of interaction compared to larger particles^37^. Particles smaller than 10 nm interact with bacteria and produce electronic effect which enhance their reactivity^38^,^39^. The particle size range of synthesized Ag/AgO NPs is 2–8 nm, hence the obtained high bacterial growth inhibition efficacy even at very low concentrations. Studies on inhibition of bacterial growth have also shown that efficacy is also influenced by shape, e.g. truncated triangular particles show complete inhibition with Ag NPs content 1 μg, spherical particles reduce bacterial growth above 12.5 μg, while a total of 50 to 100 μg of silver cause 100% inhibition of bacterial growth and for rod shaped particles it needs ~100 μg^38^.

Superior antibacterial nature of these Ag NPs over other Ag NPs is probably due to its bulk synthesis in the host framework which is directly used for antibacterial properties. While in commonly used Ag NPs which are normally get agglomerated during their use so their efficiency goes down. But in this case direct use of integrated framework **Ag/AgO@Ni-PCP** does not allow aggregation of NPs during extraction because as soon as they are extracted from the host template, their zeta potential value is less negative so highly reactive (mentioned earlier) and inhibiting the growth of bacteria. Size of Ag NPs is also a deciding factor for their antibacterial efficacy. In this case due to small sized cavities of PCP, Ag NPs are very small in diameter (2–8 nm) which have excellent antibacterial properties than commonly available Ag NPs those consist of large diameter.

It is possible that in case of Gram-positive bacteria these Ag NPs may not work so efficiently as recent study by Dasgupta *et al*. suggests that thick multilayered peptidoglycan layer in these bacteria regulates bacterial decay while Gram-negative bacteria have much thinner and single layer of peptidoglycan which is more susceptible for decay^40^.

As it has been evidenced by PXRD (Fig. 2) that the structure of the **Ni-PCP** was maintained after integration of Ag/AgO NPs within the framework. It was of interest to ascertain maximum extraction of NPs and reintegration of Ag/AgO NPs within the extracted framework. Extraction of NPs was carried out by immersion in excess ethanol, under stirring for minimum 1 h at room temperature. The colour of the extracted framework changed from dark brown to light pastel brown indicating removal of Ag/AgO NPs. Maximum removal of Ag/AgO NPs is evidenced by PXRD (Supplementary Fig. S12a) of the extracted framework which shows only a minor presence of particles. Shifting of peaks infers that the flexible 2D network undergoes distortion due to expansion and contraction of the layered structure. Ultra violet (UV) fluorescence and FTIR of the filtrate (Supplementary Figs S13 and S14) show only presence of Ag/AgO NPs. The extracted host framework was reimmersed in considered solvent under identical reaction conditions. Ag/AgO NPs were again synthesized, maintaining phase purity of the structure as evidenced by PXRD (Supplementary Fig. S12b). The colour of the extracted framework changed from light pastel brown to dark brown. This shows that this **Ni-PCP** can serve as a reusable template and or as a dry storage media for the NPs.

In conclusion it is stated that this highly flexible **Ni-PCP** is an excellent template for effectively synthesizing and storing Ag/AgO NPs at room temperature in large quantitative yields. The as-synthesized **Ag/AgO@Ni-PCP** can be used directly or the NPs can be extracted ‘on demand’ for use as remarkable potent antimicrobials to inhibit growth or kill bacteria. The extracted template can be reused to grow new batches of Ag/AgO NPs. Syntheses of NPs from different metal precursors (Au, Pd, Zn, Mn, Mg, Fe, Cu, etc) are in progress.

## Methods

### Insitu growth of Ag/AgO NPs

Host framework (**Ni-PCP**) (0.04 g, 0.02 mmol) was immersed in a methanol/water (3 ml, 2:1 v/v) solution of AgNO_3_ (0.01 g, 0.06 mmol) at room temperature for 48 h under stirring to afford a dark brownish fibrous solid product which was filtered. Colour change of the host solid is attributed to integration of Ag/AgO NPs. Filtrate was washed with excess methanol to remove any free AgNO_3_ and dried. FTIR (KBr pellet): υ = 3397 (m), 1610 (s), 1509 (s), 1363 (s) cm^−1^.

Anal. calcd. for the solid **Ag/AgO@Ni-PCP** isolated after 96 h reaction time {[Ni_3_^III^(TBIB)_2_(BTC)_2_(H_2_O)_6_]}∙2CH_3_OH∙(NO_3_)_12_∙12Ag/AgO (Ni_3_C_74_H_62_N_24_O_56_Ag/AgO_12_): C, 24.32; H, 1.71; N, 9.19. Found: C, 24.4; H, 1.74; N, 9.2. ICP data (in HNO_3_): concentration ratio of Ag/AgO:Ni = 102.28:25.65; mol ratio of Ag/Ni ≈ 4. FTIR (KBr pellet): υ = 3423.34 (m), 1604 (s), 1508 (s), 1357 (s) cm^−1^.

### Cell culture and treatment

For *Escherichia coli (E. coli*), DH5α strain and for *Salmonella typhimurium (S. typhimurium)*, TA100 strain was used for **Ag/AgO@Ni-PCP** sensitivity assay. *E. coli* strains were stored in Luria-Bertani (LB) medium containing 15% glycerol at −80 °C, and were grown in LB broth at 37 °C for 16 h under shaking at 250 rpm. *S. typhimurium* were grown in growth media (provided in Xenometrics kit for mutagenesis assay: “Ames MPF™ Penta”) with added ampicillin (50 mg/ml) at 37 °C for 16 h under shaking at 250 rpm. *E. coli* (DH5α) and *S. typhimurium* (TA100) were assessed for their cell growth by measuring their turbidity at 600 nm (OD600). OD > 2.5 at 600 nm was checked for sufficient growth and further **Ag/AgO@Ni-PCP** treatment. Bacterial cultures (OD600 > 2.5) were sub-cultured in LB broth and growth media with different concentration of **Ag/AgO@Ni-PCP** and then incubated at 37 °C for 16 h under shaking at 250 rpm. OD values were taken after this step. Suspended concentrations of **Ag/AgO@Ni-PCP** used for assay were 300, 200, 100, 60, 50, 40 and 20 μg/ml corresponding to calculated equivalent of 96, 64, 32, 21.3, 16, 13.3 and 6.6 μg/ml Ag/AgO NPs respectively. Triplicate samples from each treatment were obtained for the determination of mean values and standard deviations.

### Statistical analysis

Data was plotted and statistically analyzed using Prism software program (GraphPad Software, USA).

### Plate method

Escherichia coli bacteria (*E. coli*) were inoculated in the LB broth for 16 h. to obtain a primary culture of 0.7 O.D. The bacterial culture was then divided into two parts. One was mixed with **Ag/AgO@Ni-PCP** to achieve a final concentration of 20 μg/ml and the other part was used as a negative control. Both the cultures were diluted using LB broth with a dilution factor of 1:1 and 1:4 ratio and were spread on the LB agar plates immediately after mixing the NPs and the time point was noted as 0 h. The LB agar experimental and control plates were then incubated for 16 h at 37 °C and the bacterial growth was recorded. All the agar plates were incubated for 16 h at 37 °C. All the experiments were performed in triplicates.

## Additional Information

**How to cite this article:** Agarwal, R. A. *et al*. Ag/AgO Nanoparticles Grown via Time Dependent Double Mechanism in a 2D Layered Ni-PCP and Their Antibacterial Efficacy. *Sci. Rep.* **7**, 44852; doi: 10.1038/srep44852 (2017).

**Publisher's note:** Springer Nature remains neutral with regard to jurisdictional claims in published maps and institutional affiliations.

## Supplementary Material

Supplementary Information

## References

[b1] Vermoortele F. . p-xylene-selective metal–organic frameworks: a case of topology-directed selectivity. J. Am. Chem. Soc. 133, 18526–18529 (2011). 22022950 10.1021/ja207287h

[b2] Furukawa H. . Ultrahigh porosity in metal-organic frameworks. Science 329, 424–428 (2010). 20595583 10.1126/science.1192160

[b3] Min K. S. & Suh M. P. Silver(I)−polynitrile network solids for anion exchange: anion-induced transformation of supramolecular structure in the crystalline state. J. Am. Chem. Soc. 122, 6834–6840 (2000).

[b4] Lee J. Y. . Metal–organic framework materials as catalysts. Chem. Soc. Rev. 38, 1450–1459 (2009). 19384447 10.1039/b807080f

[b5] Ma L., Abney C. & Lin W. Enantioselective catalysis with homochiral metal– organic frameworks. Chem. Soc. Rev. 38, 1248–1256 (2009). 19384436 10.1039/b807083k

[b6] Allendorf M. D., Bauer C. A., Bhakta R. K. & Houk R. J. T. Luminescent metal–organic frameworks. Chem. Soc. Rev. 38, 1330–1352 (2009). 19384441 10.1039/b802352m

[b7] Weber I. T. . High photoluminescent metal-organic frameworks as optical markers for the identification of gunshot residues. Anal. Chem., 83, 4720–4723 (2011). 21585195 10.1021/ac200680a

[b8] Kawano M. & Fujita M. Direct observation of crystalline-state guest exchange in coordination networks. Coord. Chem. Rev. 251, 2592–2605 (2007).

[b9] Wang Z. & Cohen S. M. Postsynthetic modification of metal–organic frameworks. Chem. Soc. ReV. 38, 1315–1329 (2009). 19384440 10.1039/b802258p

[b10] Heiz U. & Landman U. (eds). Nanocatalysis. Springer (2007).

[b11] Anker J. N. . Biosensing with plasmonic nanosensors. Nature Mater 7, 442–453 (2008). 18497851 10.1038/nmat2162

[b12] Jin R. . Nanoparticle growth through plasmon excitation. Nature 425, 487–490 (2003). 14523440 10.1038/nature02020

[b13] Maier S. A. . Plasmonics—a route to nanoscale optical devices. Adv. Mater. 13, 1501–1505 (2001).

[b14] Noginov M. A. . Demonstration of a spaser-based nanolaser. Nature 460, 1110–1112 (2009). 19684572 10.1038/nature08318

[b15] Atwater H. A. & Polman A. Plasmonics for improved photovoltaic devices. Nature Mater 9, 205–213 (2010). 20168344 10.1038/nmat2629

[b16] Arvizo R. R. . Intrinsic therapeutic applications of noble metal nanoparticles: past, present and future. Chem. Soc. Rev. 41, 2943–2970 (2012). 22388295 10.1039/c2cs15355f PMC3346960

[b17] Lue J.-T. A review of characterization and physical property studies of metallic nanoparticles. J. Phys. Chem. Solids. 62, 1599–1612 (2001).

[b18] Kelly K. L., Coronado E., Zhao L. L. & Schatz G. C. The optical properties of metal nanoparticles: the influence of size, shape, and dielectric environment. J. Phys. Chem. B, 107, 668–677 (2003).

[b19] Schröder F. . Ruthenium nanoparticles inside porous [Zn4O(bdc)3] by hydrogenolysis of adsorbed [Ru(cod)(cot)]: a solid-state reference system for surfactant-stabilized ruthenium colloids. J. Am. Chem. Soc. 130, 6119–6130 (2008). 18402452 10.1021/ja078231u

[b20] Wang C., Dekrafft K. E. & Lin W. Pt nanoparticles@photoactive metal-organic frameworks: efficient hydrogen evolution via synergistic photoexcitation and electron injection. J. Am. Chem. Soc. 134, 7211–7214 (2012). 22486151 10.1021/ja300539p

[b21] Jiang H.-L. . Au@ZIF-8: CO oxidation over gold nanoparticles deposited to metal−organic framework. J. Am. Chem. Soc. 131, 11302–11303 (2009). 19637919 10.1021/ja9047653

[b22] Saikia M. & Saikia L. Palladium nanoparticles immobilized on an amine-functionalized MIL-101(Cr) as a highly active catalyst for oxidative amination of aldehydes. RSC Adv. 6, 14937–14947 (2016).

[b23] Hermes S., Schröder F., Amirjalayer S., Schmid R. & Fischer R. A. Loading of porous metal–organic open frameworks with organometallic CVD precursors: inclusion compounds of the type [LnM]a@MOF-5. J. Mater. Chem. 16, 2464–2472 (2006).

[b24] Hermes S. . Metal@MOF: loading of highly porous coordination polymers host lattices by metal organic chemical vapor deposition. Angew. Chem., Int. Ed. 44, 6237–6241 (2005). 10.1002/anie.200462515 16130164

[b25] Bak W., Kim H. S., Chun H. & Yoo W. C. Facile synthesis of metal/metal oxide nanoparticles inside a nanoporous carbon matrix (M/MO@C) through the morphology-preserved transformation of metal–organic framework. Chem. Commun. 51, 7238–7241 (2015). 10.1039/c5cc01701g 25813137

[b26] Moon H. R., Kim J. H. & Suh M. P. Redox-active porous metal–organic framework producing silver nanoparticles from AgI Ions at room temperature. Angew. Chem. 117, 1287–1291 (2005) *Angew. Chem. Int. Ed.* **44**, 1261-1265 (2005). 10.1002/anie.200461408 15645526

[b27] Suh M. P., Moon H. R., Lee E. Y. & Jang S. Y. A redox-active two-dimensional coordination polymer: preparation of silver and gold nanoparticles and crystal dynamics on guest removal. J. Am. Chem. Soc. 128, 4710–4718 (2006). 16594708 10.1021/ja056963l

[b28] Shimomura S., Matsuda R., Tsujino T., Kawamura T. & Kitagawa S. TCNQ dianion-based coordination polymer whose open framework shows charge-transfer type guest inclusion. J. Am. Chem. Soc. 128, 16416–16417 (2006). 17177347 10.1021/ja0660047

[b29] Agarwal R. A. & Mukherjee S. Two-dimensional flexible Ni(II)-based porous coordination polymer showing single-crystal to single-crystal transformation, selective gas adsorption and catalytic properties. Polyhedron 105, 228–237 (2016).

[b30] Zhao J. . BaFe_12_O_19_-chitosan Schiff-base Ag (I) complexes embedded in carbon nanotube networks for high-performance electromagnetic materials. Scientific Reports. 5, 12544, doi: 10.1038/srep12544. PMC4649749 26218269

[b31] Dubnika A., Loca D., Reinis A., Kodols M. & Berzina-Cimdina L. Impact of sintering temperature on the phase composition and antibacterial properties of silver-doped hydroxyapatite. Pure Appl. Chem. 85, 315–462 (2013).

[b32] Yue Z. R. . Adsorption of precious metal ions onto electrochemically oxidized carbon fibers. Carbon 37, 1607–1618 (1999).

[b33] Reddy P. N., Reddy M. H. P., Pierson J. F. & Uthanna S. Characterization of silver oxide films formed by reactive RF sputtering at different substrate temperatures. ISRN Optics. 2014, Article ID 684317, 10.1155/2014/684317.

[b34] Material Measurement Laboratory (MML) NIST, U.S. Department of Commerce (2007).

[b35] Melendrez M. F., Cardenas G. & Arbiol J. Synthesis and characterization of gallium colloidal nanoparticles. J. Colloid Interface Sci. 346, 279–287 (2010). 20378122 10.1016/j.jcis.2009.11.069

[b36] Rycenga M. . Controlling the synthesis and assembly of silver nanostructures for plasmonic applications. Chem Rev. June 8, 111(6), 3669–3712 (2011). 21395318 10.1021/cr100275d PMC3110991

[b37] Morones J. R. . The bactericidal effect of silver nanoparticles. Nanotechnology. 16, 2346–2353 (2005). 20818017 10.1088/0957-4484/16/10/059

[b38] Pal S., Tak Y. K. & Song J. M. Does the antibacterial activity of silver nanoparticles depend on the shape of the nanoparticle? a study of the gram-negative bacterium escherichia coli. Appl. Environ. Microbiol. 73, 1712–1720 (2007). 17261510 10.1128/AEM.02218-06 PMC1828795

[b39] Raimondi F., Scherer G. G., Kötz R. & Wokaun A. Nanoparticles in energy technology: examples from electrochemistry and catalysis. Angew. Chem. Int. Ed. 44, 2190–2209 (2005). 10.1002/anie.200460466 15776488

[b40] Dasgupta S., Gunda N. S. K. & Mitra S. K. Evaluation of the antimicrobial activity of Moringa oleifera seed extract as a sustainable solution for potable water. RSC Adv. 6, 25918–25926 (2016).

