# Supplementary material for: Ag/AgO Nanoparticles Grown via Time Dependent Double Mechanism in a 2D Layered Ni-PCP and Their Antibacterial Efficacy
Source: Sci Rep. 2017 Mar 21;7:44852. doi: 10.1038/srep44852 (PMC5359589; doi:10.1038/srep44852)
Supplement: Supplementary Information [file srep44852-s1.pdf]

Ag/AgO Nanoparticles Grown via Time Dependent Double Mechanism in a 2D Layered Ni-PCP and Their Antibacterial Efficacy

Rashmi A. Agarwal[1*], Neeraj K. Gupta[2], Rajan Singh[3a], Shivansh Nigam[3b] & Bushra Ateeq[3]

[1]*Department of Chemistry, Indian Institute of Technology Kanpur, 208016, India*

[2]*Department of Mechanical Engineering, Indian Institute of Technology Kanpur, 208016, India*

[3]*Department of Biological Science and Bioengineering, Indian Institute of Technology Kanpur, 208016, India*

Supplementary Information

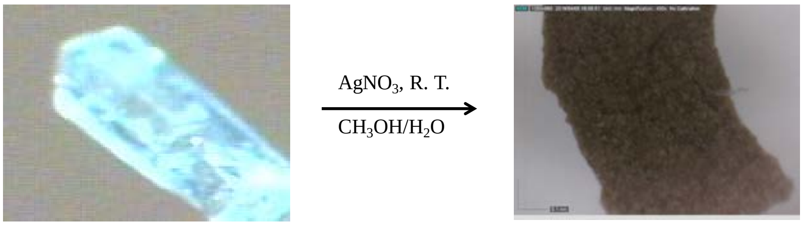

(a) (b)

Figure S1. Surface morphology before and after growth of NPs. (a) Single crystal image of host template (**Ni-PCP**). **(b)** Dinomicroscope image of host solid isolated after immersion in CH_3OH/H_2O solution of $AgNO_3$ at room temperature for 48 h.

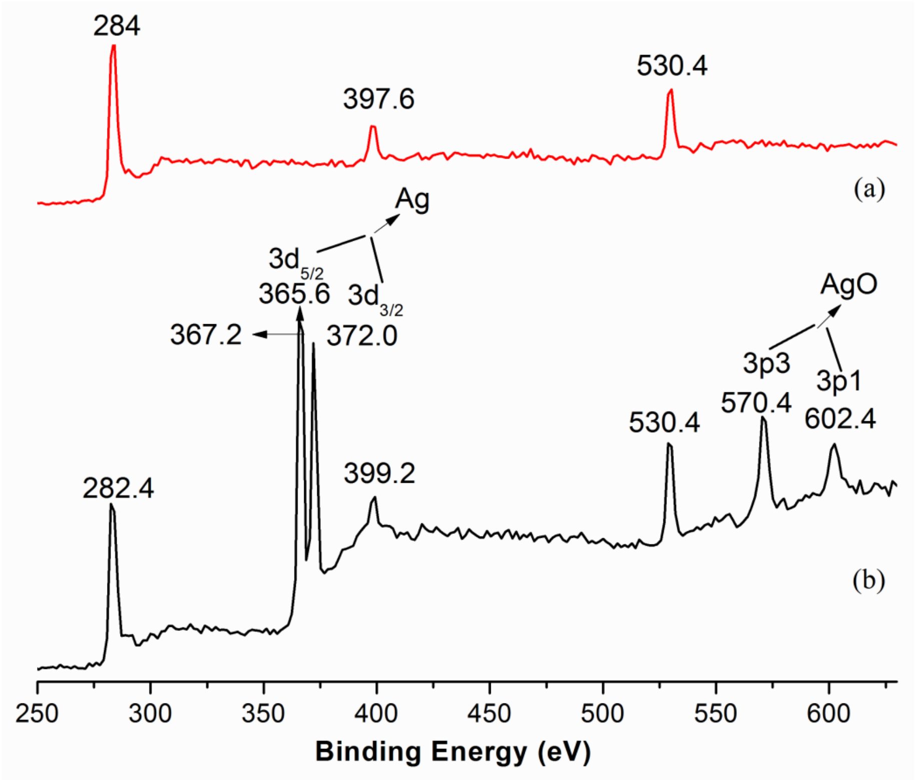

Figure S2. X-ray photoelectron spectra. (a) Host framework **Ni-PCP**. **(b) Ag/AgO@Ni-PCP** where binding energies corresponding to Ag/AgO NPs are clearly seen.

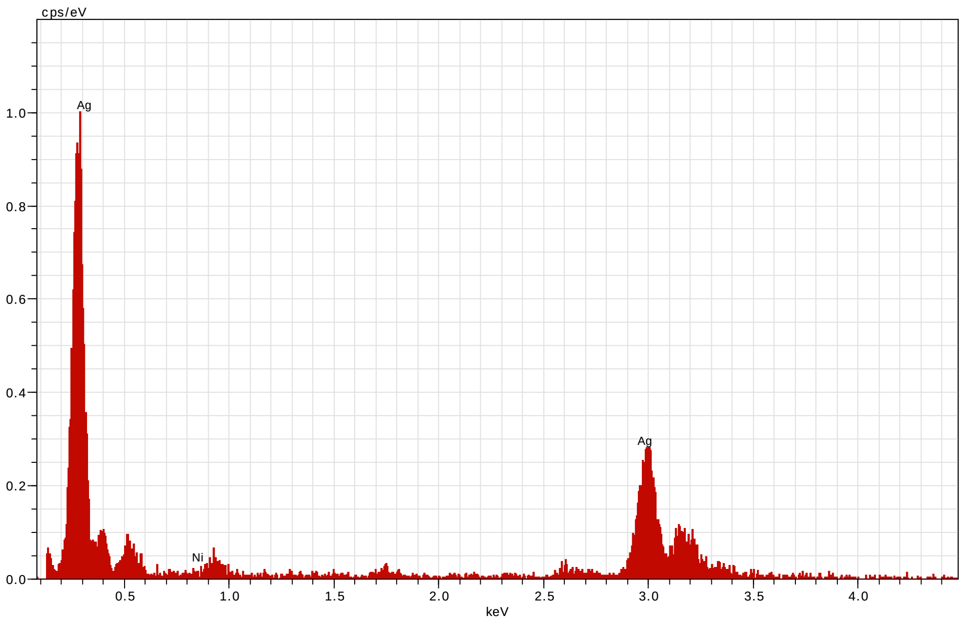

Figure S3. Energy dispersive spectroscopic data of Ag/AgO@Ni-PCP showing coexistence of both metals coordinated Ni ions as well as synthesized Ag metal.

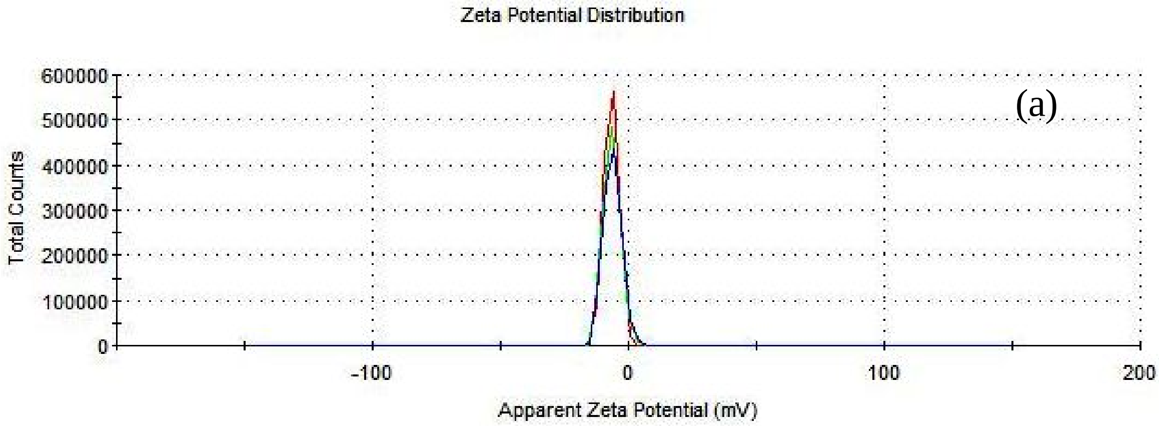

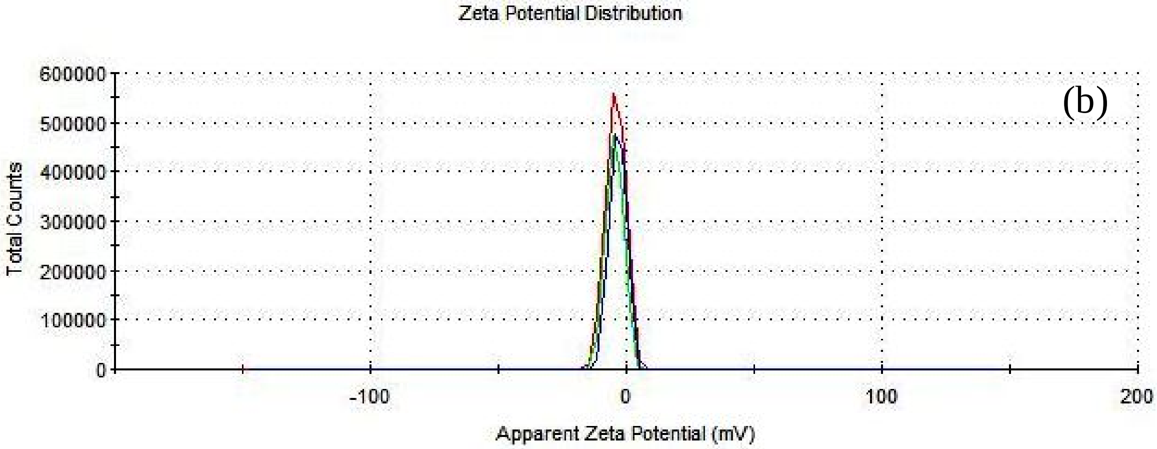

Figure S4. Zeta potential (−6.72 to −4.12 mV) of extracted NPs from two independent suspensions of Ag/AgO@Ni-PCP showing that extracted NPs are highly reactive.

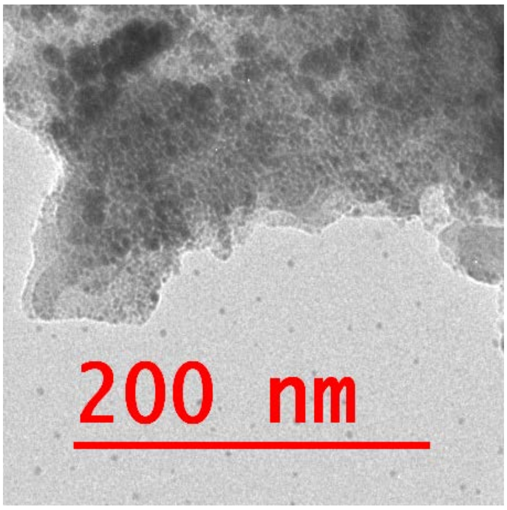

(a)

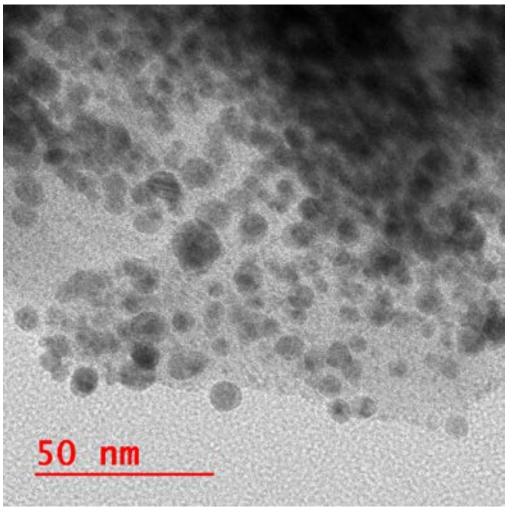

(b)

Figure S5. HRTEM images of Ag/AgO NPs encapsulated framework synthesized after 96 h reaction time. (a) Ag/AgO NPs released from **Ag/AgO@Ni-PCP** due to sonication during sample preparation. **(b)** Shape and size (2-10 nm) of Ag/AgO NPs along with slightly agglomeration.

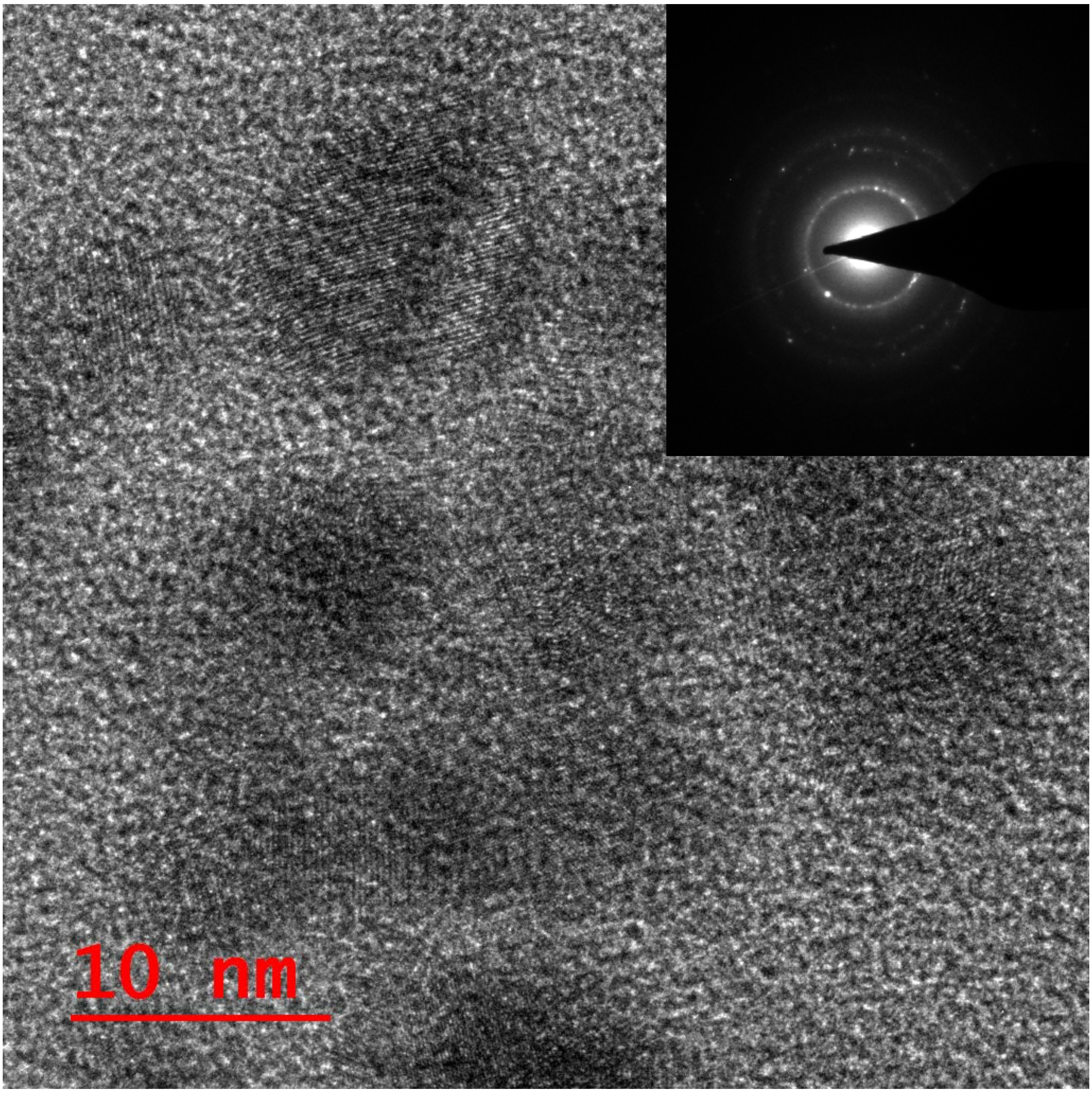

Figure S6. HRTEM image of Ag/AgO@Ni-PCP showing crystalline planes of synthesized Ag/AgO NPs after 48 h reaction time.

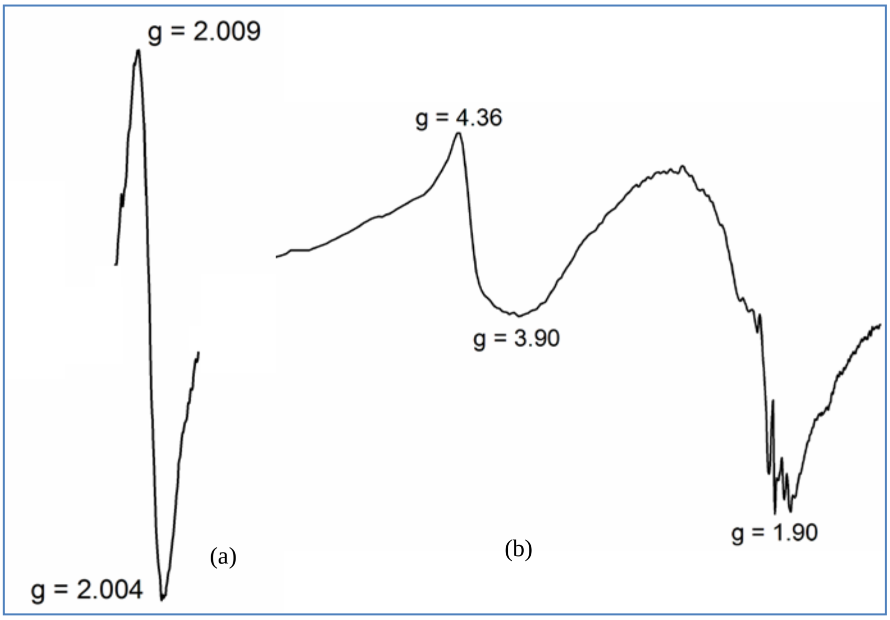

Figure S7. EPR spectrum of Synthesized Ag/AgO@Ni-PCP. (a) After 48 h reaction time **(b)** After 96 h reaction time.

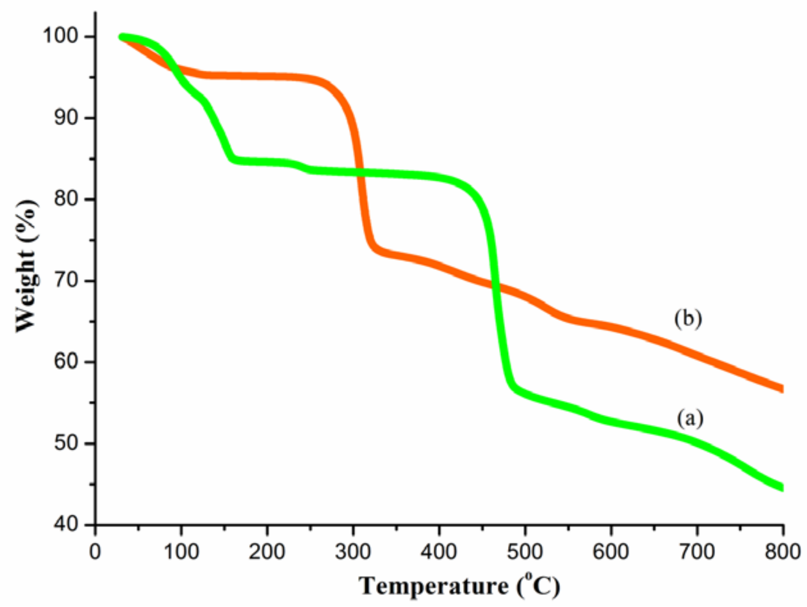

Figure S8. Thermogravimetric analysis plots. (a) Ni-PCP host framework displaying excellent stability till 420 ºC temperature. **(b) Ag/AgO@Ni-PCP** showing less thermal stability due to the catalytic activity of Ag/AgO NPs synthesized after 48 h reaction time in methanol/water solvent.

Table 1. Weight loss by thermogravimetry.

	Weight Loss (wt%)				
	1st Step	2nd Step	3rd Step	4th Step	5th Step
**Ni-PCP**	4.0 (40 - 96 ºC)	3.3 (96-118 ºC)	7.5 (118-161 ºC)	1.5 (161-256 ºC)	26.6 (420-490 ºC)
**Ag/AgO@Ni-PCP**	4.4 (34-120)	-	-	-	21.9 (267-329)

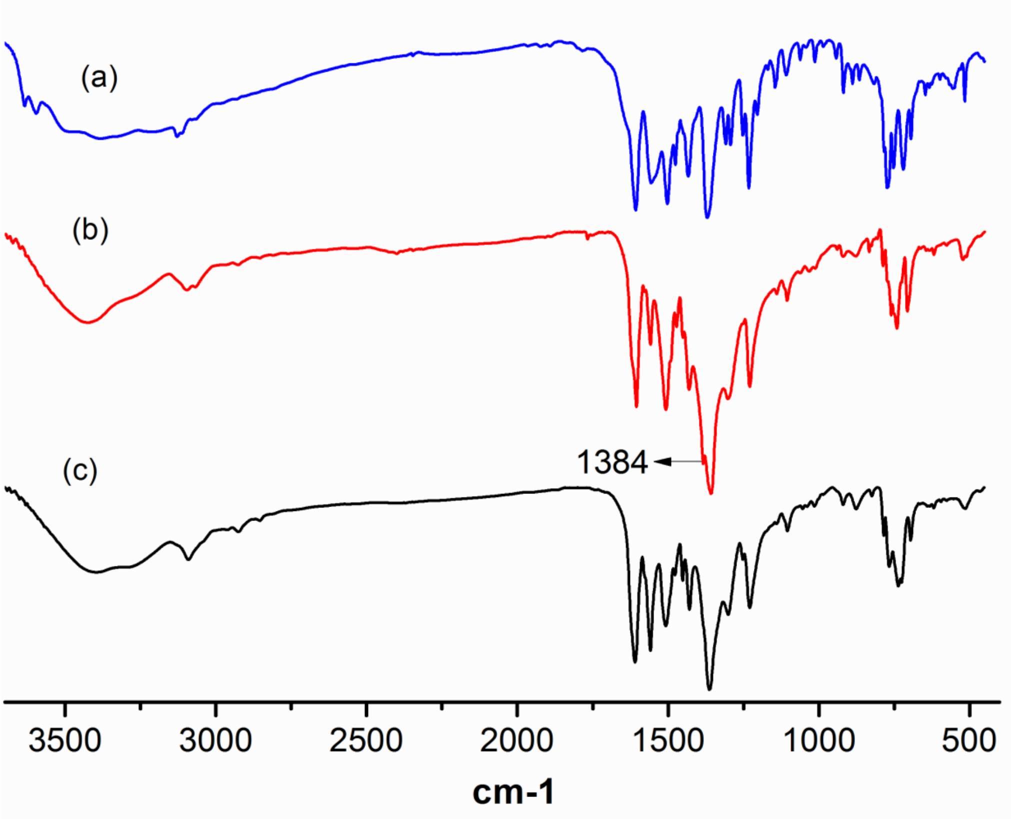

Figure S9. FTIR spectra. (a) Ni-PCP (host framework). **(b)** Reaction after 96 h showing NO_3^- anion peak in **Ag/AgO@Ni-PCP** which is generated due to compensate extra positive charge of Ni(III). **(c) Ag/AgO@Ni-PCP** after 48 h of reaction wherein slight peak shift are observed.

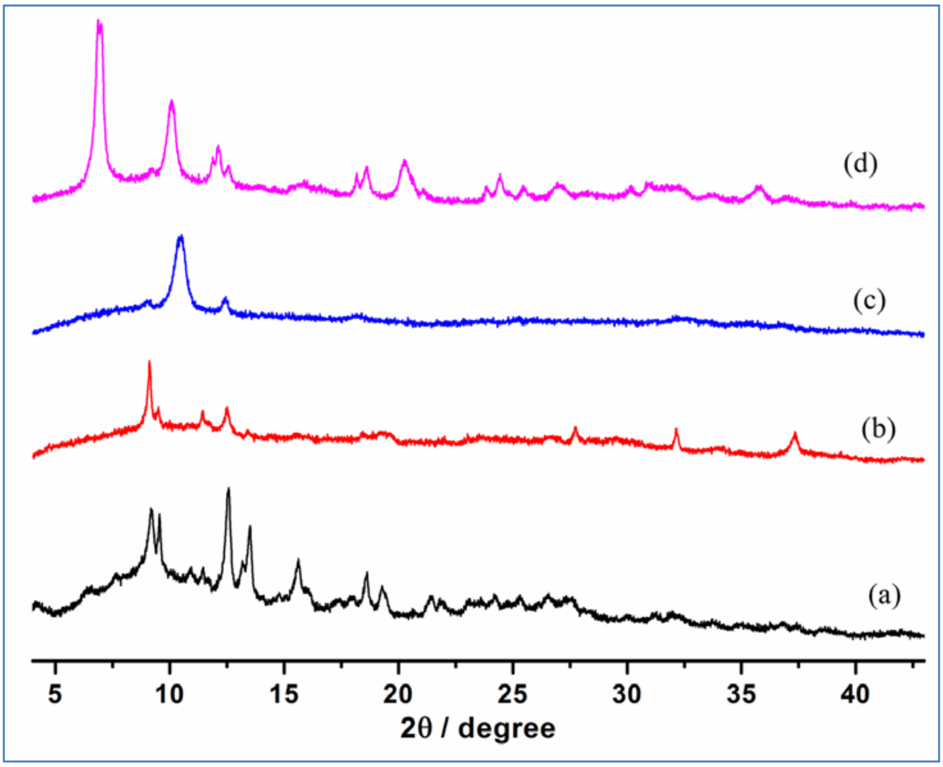

Figure S10. PXRD patterns of Ag/AgO@Ni-PCPs from reactions held at 48 h using different solvents. (a) Ag/AgO@Ni-PCP synthesized using acetonitrile as a solvent. **(b) Ag/AgO@Ni-PCP** synthesized utilizing water as a polar solvent reaction media giving rise crystalline NPs with homogeneous particle size distribution. **(c) Ag/AgO@Ni-PCP** synthesized using ethanol solvent. **(d) Ag/AgO@Ni-PCP** synthesized using methanol solvent.

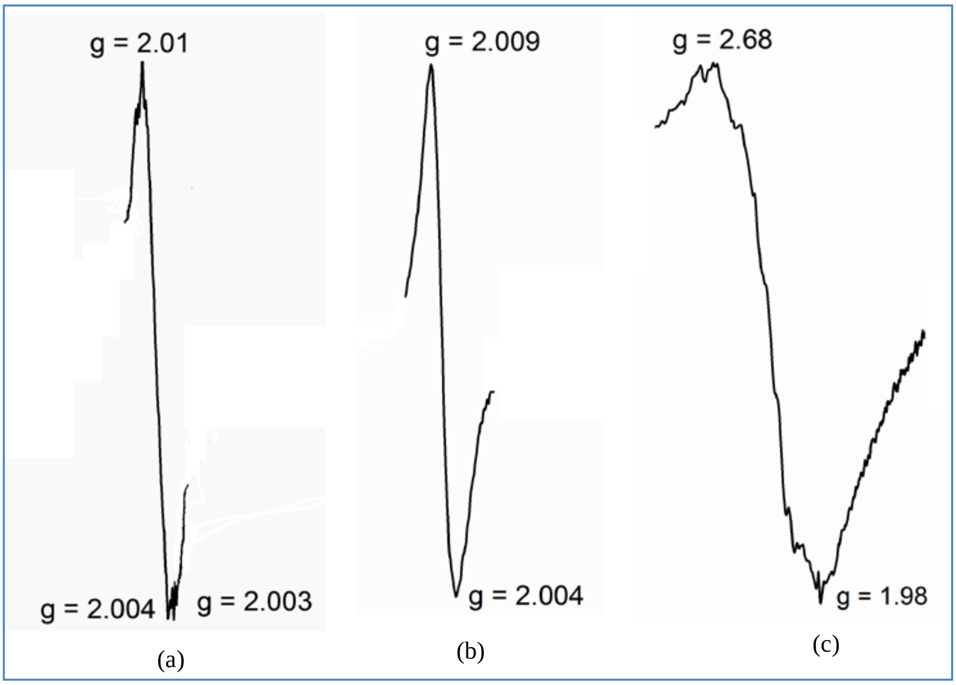

Figure S11. EPR spectrum taken of Ag/AgO@Ni-PCP synthesized in different solvents for 48 h reaction time at room temperature. (a) Methanol solvent. **(b)** Water solvent. **(c)** Acetonitrile solvent.

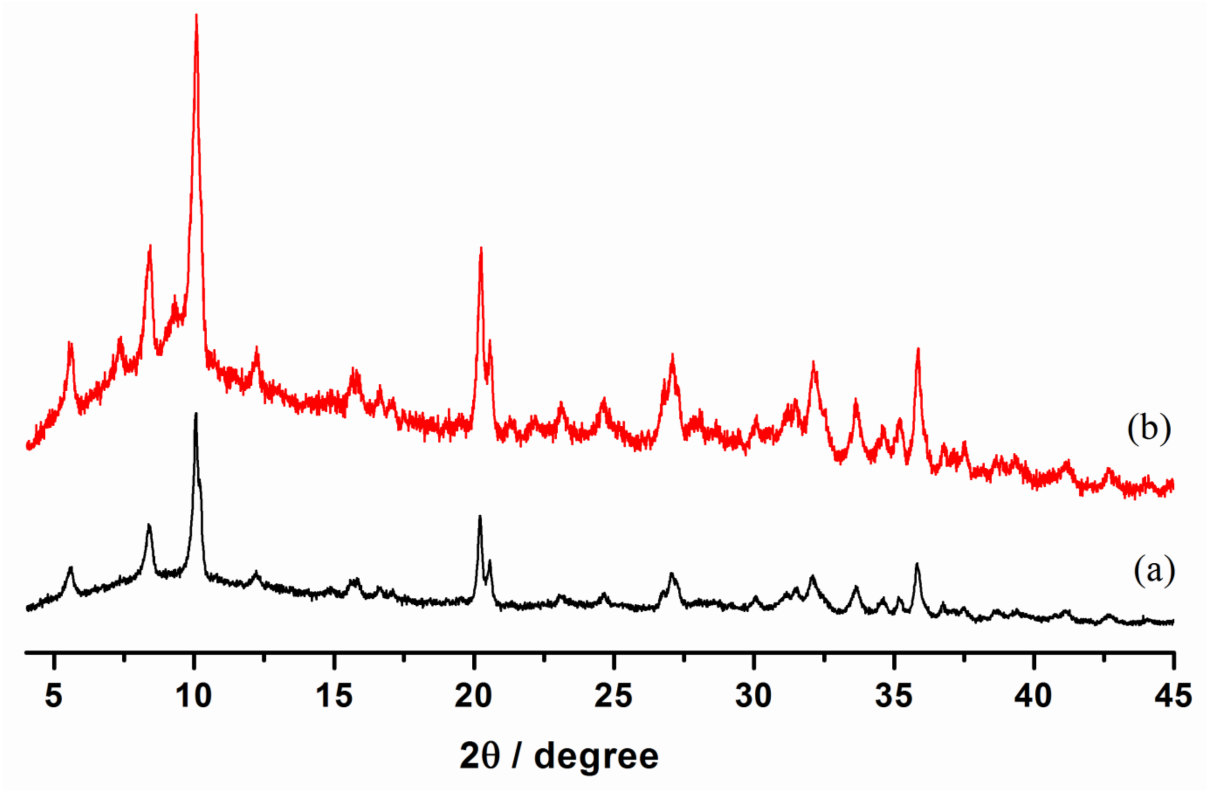

Figure S12. PXRD patterns. (a) Extracted framework after Ag/AgO NPs removal from **Ag/AgO@Ni-PCP** in ethanol solment under stirring for 1 h. **(b)** Framework after regrowth of Ag/AgO NPs for 48 h reaction time at room temperature in methanol/water solvent medium.

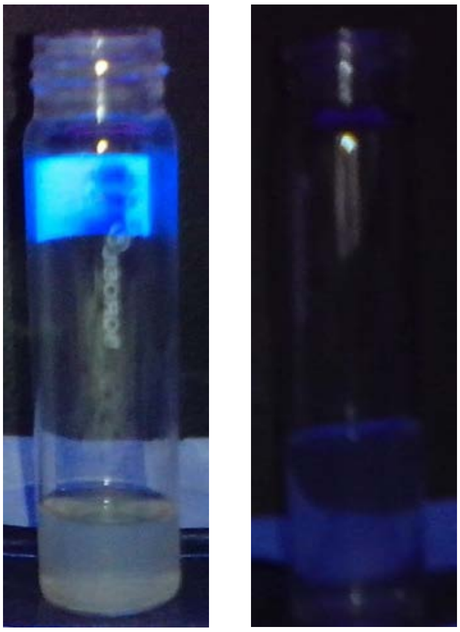

(a) (b)

Figure S13. UV fluorescence images. (a) No fluorescence observed on immidiate immersion of **Ag/AgO@Ni-PCP** in ethanol solvent. **(b)** Blue fluorescence observed after immersion of **Ag/AgO@Ni-PCP** in ethanol solvent under stirring for 1 h due to Ag/AgO NPs removal.

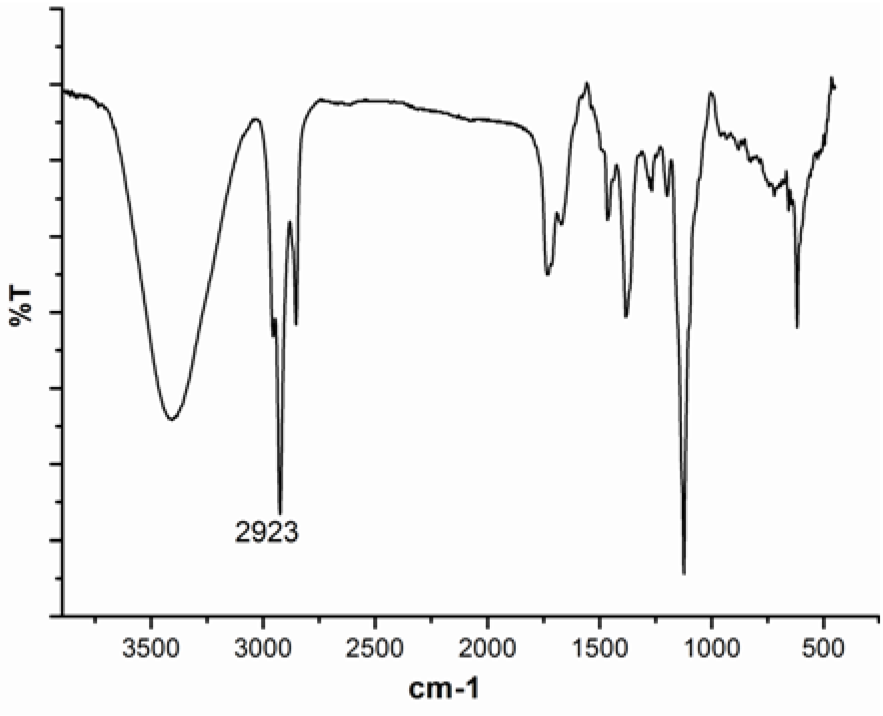

Figure S14. FTIR spectra of ethanol solvent in which Ag/AgO NPs are extracted from Ag/AgO@Ni-PCP under stirring for 1 h at room temperature.
